# Thresholds for spring freeze: measuring risk to improve predictions in a warming world

**DOI:** 10.1111/nph.70453

**Published:** 2025-08-07

**Authors:** Erica Kirchhof, Francisco Campos‐Arguedas, Nadia Soledad Arias, Al P. Kovaleski

**Affiliations:** ^1^ Department of Plant and Agroecosystem Sciences University of Wisconsin‐Madison Madison WI 53706 USA; ^2^ Instituto de Biociencias de la Patagonia (INBIOP) Consejo Nacional de Investigaciones Científicas y Técnicas (CONICET) and Universidad Nacional de la Patagonia San Juan Bosco (UNPSJB) Comodoro Rivadavia 90000 Argentina; ^3^ Departamento de Biología y Ambiente, Facultad de Ciencias Naturales y Ciencias de la Salud Universidad Nacional de la Patagonia San Juan Bosco (UNPSJB) Comodoro Rivadavia 90000 Argentina

**Keywords:** cold hardiness, frost risk, phenological stage, phenology, spring freeze, spring frost, temperature threshold

## Abstract

Plant distribution and productivity are shaped by environmental stressors, particularly freezing events in extra‐tropical regions. In early spring, a progressive loss of cold hardiness with phenological development leaves emerging tissues vulnerable to freezing events. In many regions, climate warming is advancing phenology to a greater degree than the date of the last spring freeze, increasing the period of vulnerability to spring freezes. Studies describing critical temperatures at which spring freeze damage occurs are numerous and diverse in both the system studied and type of thresholds used (empirical vs model). Here, we review trends in previously reported thresholds for spring freeze damage across a range of plant groups (row crops, fruit trees, and forest species) over the course of phenological development in spring, and analyze potential sources of variation causing discrepancies between empirically determined and model‐derived thresholds. Our analysis shows consistent reporting of higher (less‐hardy) thresholds in model‐based studies when compared with empirical measurements. These differences highlight a need to improve model estimations of damage by accounting for both microclimatic complexities (humidity, topography, wind speed, etc.) and physiological considerations (phenology and species) that influence the translatability between precise, empirically measured thresholds and critical temperatures used to describe damage in the field.

## Introduction

Environmental factors shape global plant distribution, and low temperature is one of the most critical abiotic stressors (Inouye, 2022). Below freezing temperatures set ecological boundaries, limit ranges, and inflict economic losses for both natural and agricultural ecosystems (Chamberlain & Wolkovich, 2021; Körner, 2021; Drepper *et al*., 2022; Qian *et al*., 2022). Because spring marks the transition from dormant, cold‐hardy states (e.g. dormant buds and seeds) to less cold‐hardy visible and active growth, freezing events during this period are critical in determining the success of both the immediately upcoming and future growing seasons (Parker *et al*., 2021; Baumgarten *et al*., 2023; Wang *et al*., 2025). There is also evidence that spring freezes can be more important than mid‐winter low temperature damage to define the cold range edge of distributions (Körner *et al*., 2016). In an agricultural context, ‘late spring freezes’ are a frequent challenge for species introduced in areas beyond their optimum climate niche. However, greater advancement of spring phenological development relative to the date of last frost due to climate change is increasing the frequency of this issue within natural ecosystems as well (Liu *et al*., 2018; Sgubin *et al*., 2018; Pfleiderer *et al*., 2019; Chamberlain & Wolkovich, 2021). Therefore, understanding the abilities of plants to survive these low‐temperature stresses during spring is a critical aspect of adaptation and an important determinant of future species distributions.

Frosts and freezes are different events based on climatology definitions. Generally, freezes refer to the occurrence of temperatures at or below 0°C, the equilibrium freezing point of water. By contrast, frosts refer to events when ice is formed on surfaces through sublimation, which requires both the surfaces' temperature and the dew point to be below 0°C. Most studies that aim to identify low‐temperature damage during spring use air temperature as a proxy for quantifying the tissue temperature threshold at which the damage occurs (Meier *et al*., 2018; Sgubin *et al*., 2018; Mura *et al*., 2022), which facilitates some level of comparison across datasets and studies. The formation of frost, however, is affected by other environmental aspects such as humidity, wind, and surface conditions. As such, it can be difficult to infer the occurrence of frost simply based on the recorded air temperature (Sentelhas *et al*., 1995). Recognizing these nuances is essential to improving our ability to understand and accurately report and describe low‐temperature damage. Because of the existing lack of clarity and for the sake of continuity, here we refer to the low temperature events causing damage in spring from both freezes (temperature ≤ 0°C) and classically defined frosts (ice deposition) as ‘spring freezes’.

The magnitude, severity, and timing of spring freezes can have a profound impact on a plant's ability to survive in a given region over time (Körner *et al*., 2016; Baranger *et al*., 2024). Plant species that maintain living structures during winter (e.g. woody perennials, bulbs) survive low temperatures by developing cold hardiness, acquired during autumn and early winter months (acclimation) and gradually lost in late winter to early spring (deacclimation). During the deacclimation phase, newly expanding tissues are particularly vulnerable to spring freeze events (Chamberlain *et al*., 2019; North & Kovaleski, 2024). A brief return to freezing after unseasonal warmth (a phenomenon known as ‘false spring’) can therefore be lethal to developing tissues (Korstian, 1921; Hufkens *et al*., 2012; Chamberlain *et al*., 2019; Montgomery *et al*., 2020; Kovaleski, 2024). From an ecological perspective, late spring freezes can disrupt plant–pollinator synchrony, delay succession, increase vulnerability to secondary stressors such as herbivory and pathogens, and reduce terrestrial carbon uptake (Inouye, 2008; Willmer, 2012; Forrest, 2015). In agricultural systems, freeze damage to vegetative tissue delays seasonal growth and indirectly decreases yield (Carter, 1995; Elmore & Doupnik Jr., 1995; Meyer & Badaruddin, 2001; Li *et al*., 2015), while freeze damage to reproductive tissues can lead to substantial direct reductions in yield (Sgubin *et al*., 2018; Guo *et al*., 2024; Kim *et al*., 2025). Globally, many such examples have been well documented: a 2007 extended late spring freeze event caused substantial reductions in yield for many fruit and nut crops in the United States (Gu *et al*., 2008); an April 2017 freeze caused significant damage to both forest trees and crops throughout Switzerland and Germany (Vitasse *et al*., 2018); and a 2018 spring freeze devastated tea plantations in the Shaanxi Province of China (Tang *et al*., 2023), to name a few.

Late spring freezes are not uncommon in temperate and boreal climates, and native and well‐adapted species maintain enough tissue hardiness to survive these events (Sierra‐Almeida *et al*., 2009; Neuner, 2014). However, as climate warming causes phenological phases to advance more rapidly than the date of the last spring freeze, the likelihood that young, developing tissues are exposed to brief periods of potentially damaging cold temperatures increases (Chamberlain *et al*., 2019; Chamberlain & Wolkovich, 2021; Lamichhane, 2021). Thus, many studies have been performed to identify the critical temperature thresholds at which developing plant tissues are most vulnerable, as well as to predict how the risk of spring freeze events may shift under future climate scenarios (Marino *et al*., 2011). Additional microclimatic and genotypic nuances further complicate these predictions. As a result, there are existing, potentially critical discrepancies between general climate models using simple damage thresholds and the species‐specific responses that ultimately determine survival and yield.

Studies that aim to quantify spring freezes are numerous and exist for a variety of plant groups, and as a result, may consider the relative importance of different factors. For example, studies focused on quantifying spring freezes in relation to shifting climatic conditions ostensibly rely on defining the patterns in atmospheric conditions that underlie these events (e.g. occurrence of air temperature ≤ 0°C (Inouye, 2008; Marquis *et al*., 2022; Wang *et al*., 2025)). Other studies, particularly those that are concerned with characterizing the physiological implications of such low‐temperature events on plants, may find it more relevant to define precise thresholds for damage to plant tissues (Miranda *et al*., 2005; Gunes, 2006; Matzneller *et al*., 2016; Szalay *et al*., 2019). In either case, the ability to translate meteorological data into actionable risk estimates requires consistent and reliable connections to be made between atmospheric conditions leading to freezes and the potential for freeze damage in plants. Here, we review conditions leading to spring freeze events and estimations of the frequency of these events with particular attention to trends in reported hardiness thresholds across a range of studies. We then examine factors that may influence discrepancies observed and use those to suggest best practices in spring freeze damage research.

## Spring freeze in the landscape: physical considerations

**Fig. 1:**
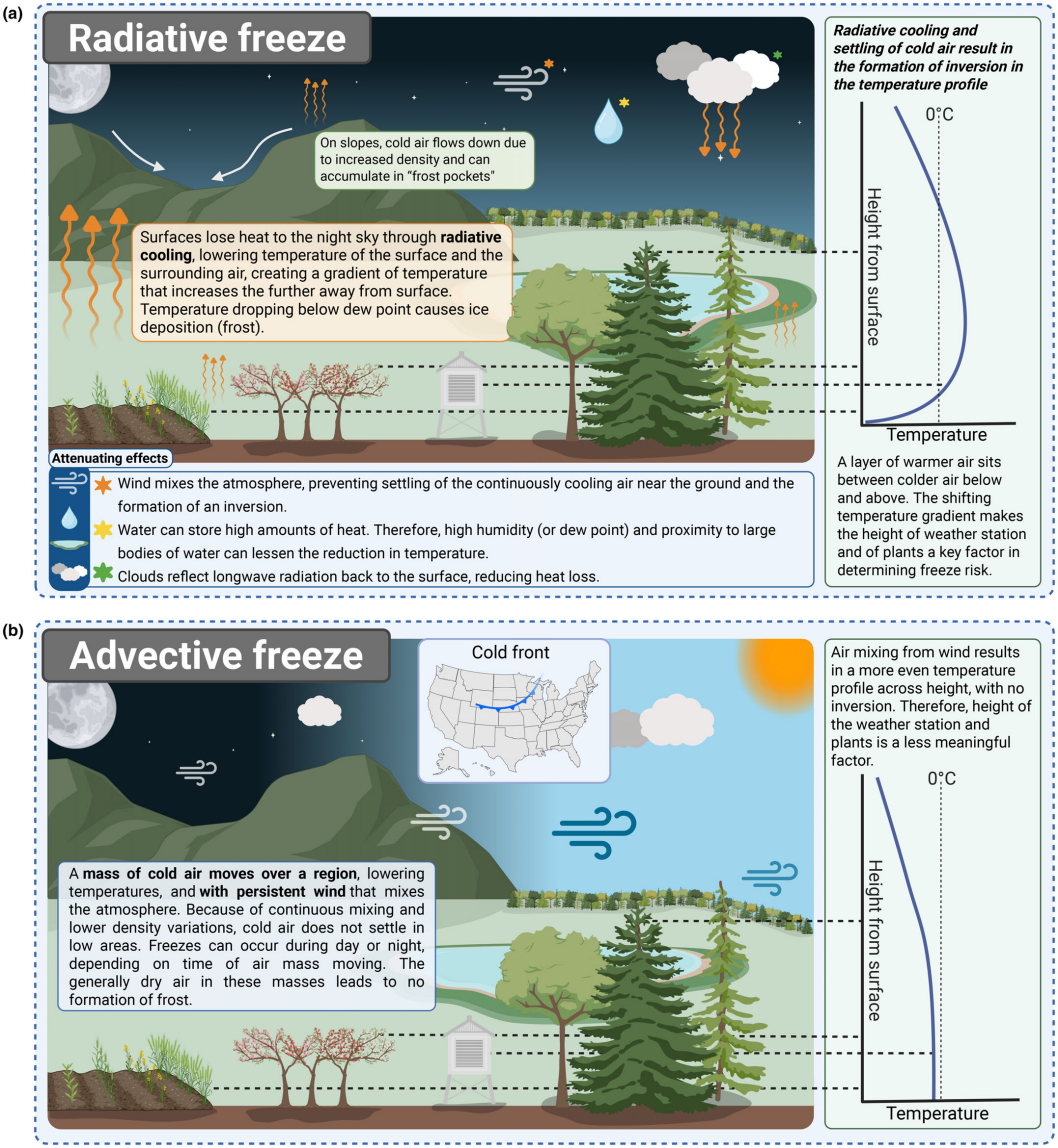
Schematic representation of different types of freezes. Depending on environmental factors, (a) radiative or (b) advective freezes may form, where each is associated with a different temperature profile. This figure was created in BioRender (BioRender.com/ib08agw).

Spring freeze events typically fall into two broad categories: radiative and advective. Radiative freezes occur under clear, calm nights when the surface of the Earth loses heat rapidly through radiation, leading to localized cooling near the ground that transfers to adjacent air (Fig. 1a). Clear nights, low wind speeds, and dry air are conditions that enhance radiative cooling and increase the likelihood of this type of freeze (Fig. 1a) (Kalma *et al*., 1992; Snyder & de Paulo, 2005; Meyer *et al*., 2024). Because these conditions usually elicit the deposition of ice, radiative freezes are usually frosts. Attenuating effects for radiative frosts therefore include cloud cover, wind, and air humidity or proximity to large bodies of water. In contrast to radiative frosts, advective frosts result from the horizontal movement of cold air masses, often accompanied by moderate to strong winds that bring freezing conditions over a large area, regardless of sky conditions or time of day (Fig. 1b). The two types of freezes therefore present different environmental conditions, which include having different temperature profiles along the vertical axis. Understanding which type of freeze is most common or likely in a location is crucial for assessing risk and providing accurate recommendations for mitigation strategies in managed environments.

Most assessments of freeze risk rely on standard air temperature measurements, typically obtained from weather stations or gridded datasets. However, these measurements often differ from the actual temperatures experienced by the plant tissue (Frustaci *et al*., 2022; Mistry *et al*., 2022; Peaucelle *et al*., 2022). Standard meteorological stations typically measure air temperature at 1.5–2 m above the ground and therefore do not always accurately capture the microclimatic conditions directly surrounding plant tissues (e.g. shrubs vs trees) (Rahn & Brown, 1971; Bootsma, 1976; Chen *et al*., 1999; Miller *et al*., 2021; Omazić *et al*., 2024). This may be particularly true for radiative frosts, where steep gradients in temperature exist with height (Fig. 1a). For example, at the grass level, temperatures have been reported to be on average 4.1°C lower than that recorded by a Stevenson screen meteorological station, with the difference being greatly influenced by wind speed, cloud cover, and dew point (Sentelhas *et al*., 1995). Similarly, taller trees have been found to experience temperatures at the top of the canopy that are greater than those near the ground (Jacobs *et al*., 1995; Rahman *et al*., 2018). Closed canopies of trees can also prevent heat loss, where only the top‐most layer is affected by radiative cooling, thus creating a warmer microclimate within the canopy (Vinod *et al*., 2023). This can protect some understory plants from spring freezes, as well as prevent some damage, particularly in densely planted agricultural environments. Therefore, when trying to accurately characterize spring freezes, differences in plant functional types should be acknowledged.

## Spring freeze damage is influenced by phenological progression

Spring freeze damage in plants is closely related to their developmental stage. Studies examining the relationship between spring freezes and developmental progression in emerging plant tissues have provided insights into how tissue vulnerability shifts throughout phenological stages (Inouye, 2008; Vitra *et al*., 2017; Sgubin *et al*., 2018; Lamichhane, 2021; Baumgarten *et al*., 2023; Muffler *et al*., 2024; Qiu *et al*., 2024; Wang *et al*., 2025). In general, as young tissues develop, they gradually lose their ability to resist spring freezes, reaching a minimum level of cold hardiness that is typically maintained or slightly enhanced once the tissue matures (Weiser, 1970; Sakai & Larcher, 1987; Neuner, 2014; Vitasse *et al*., 2014; North & Kovaleski, 2024).

Recognizing variation in phenological strategies across species adds to the context of spring freeze vulnerability patterns. Woody perennial plants have evolved two primary phenological strategies associated with their anatomy and evolutionary history. Many temperate angiosperms display a flower‐first strategy, using stored carbohydrates for early blooms, while gymnosperms and some ring‐porous angiosperms display a leaf‐first strategy, delaying flowering until after or at the same time as vegetative growth begins (Lechowicz, 1984; Kitin & Funada, 2016; Gougherty & Gougherty, 2018). However, these strategies are mostly shaped by trade‐offs. For example, gymnosperms prioritize hydraulic efficiency and continuous carbon acquisition over the risks associated with early‐season reproduction (Davies *et al*., 2013; D'Orangeville *et al*., 2022). As a result, these different evolutionary strategies have led to distinct physiological adaptations influencing plant tissue tolerance to spring freezes (Agrawal *et al*., 2004; Preston & Sandve, 2013).

The ecological implications for these differing strategies are also evident: while evergreen‐dominated boreal forests retain their leaves year‐round and can begin photosynthesis earlier in the spring and extend it later into the fall compared to deciduous angiosperms, their capacity for carbon fixation during the winter itself is severely limited by low temperatures (Givnish, 2002; Keenan *et al*., 2014; Bowling *et al*., 2024). However, because boreal evergreen forests are geographically extensive and maintain some level of photosynthetic activity during the colder shoulder seasons (Givnish, 2002; Oquist & Huner, 2003; Bowling *et al*., 2024), they play an important role in terrestrial carbon sequestration compared to temperate forests (Crous *et al*., 2022). However, evidence regarding the benefits of extended growing seasons for carbon sequestration is mixed. While longer growing seasons in temperate forests are associated with increased net carbon uptake (3.8 and 7.8 g C m^−2^ per additional day in evergreen and deciduous forests, respectively Keenan *et al*., 2014); this relationship might be constrained by earlier autumn senescence, which can offset gains from spring activity (Zohner *et al*., 2023). Moreover, evidence shows that although warmer springs extend the growing season and increase CO_2_ uptake, the additional carbon is often allocated to short‐lived tissues rather than long‐term woody biomass, limiting the potential for sustained carbon storage (Dow *et al*., 2022). Therefore, the benefits of longer growing seasons on carbon sequestration are complex and may not linearly scale under climate warming. As temperature influences the end of winter dormancy and drives spring phenology, coupling the physiological basis of these strategies with spring freeze risk would be critically informative in Earth‐system models' projections of carbon acquisition in future climate scenarios.

Pollination syndromes can also affect phenological timing, especially flowering phenology, with implications for freeze risk in temperate angiosperms. Some studies suggest that insect‐pollinated species tend to flower later than wind‐pollinated (Dowding, 1987; Wang *et al*., 2020), potentially as a strategy to synchronize with pollinators (Memmott *et al*., 2007; Tachiki *et al*., 2010).

While our analysis focuses on tissue‐level susceptibility and phenological timing, it is also important to recognize that spring freeze intersects with broader ecological dynamics. For example, early‐flushing species may gain competitive advantages in resource acquisition or pollination at the cost of higher freeze exposure – an example of a classic tradeoff influencing community structure (Lenz *et al*., 2013; Vander Mijnsbrugge & Moreels, 2020; Song *et al*., 2021). These phenological strategies can shape competitive hierarchies and contribute to community assembly by filtering species based on their tolerance to climatic extremes and their ability to recover from episodic damage (Lenz *et al*., 2013; CaraDonna & Bain, 2016).

Susceptibility to spring freezes is closely linked to phenological timing, making it critical to describe the developmental stage at which damage occurs. However, historical descriptions of phenological progression were often broad and qualitative (e.g. ‘flowering’, ‘leaf unfolding’, ‘seedling stage’) (Harrington, 1936; Leopold & Jones, 1947), making precise comparisons across studies challenging. In response, several phenological indices were developed for a variety of plants and tissues, which assign numerical categories to descriptions of phenological stage (Klemm, 1937; Soenen, 1952; Krutzsch, 1973; Zadoks *et al*., 1974). Perhaps the best known and most widely adopted of these indices is the BBCH (Biologische Bundesanstalt, Bundessortenamt und Chemische Industrie) scale, which categorizes key phenological stages – such as shoot emergence, budbreak, and flowering – using a two‐digit code that can be further subdivided for greater precision (e.g. 10% flowering, 50% flowering, green tips visible) (Weber & Bleiholder, 1990). BBCH scales have since been developed in detail for a wide variety of agriculturally relevant plants, such as legumes (Weber & Bleiholder, 1990; Lancashire *et al*., 1991), cereals (Lancashire *et al*., 1991), grapevines (Lorenz *et al*., 1995), pome fruits, and stone fruits (Meier Graf *et al*., 1994), and to a more limited extent for forest woody perennial species (Finn *et al*., 2007).

Due to its standardized structure and broad applicability, the BBCH scale is a valuable tool that should be used in studies of spring freeze, which allows for analyzing patterns across species and locations in different studies. It is important to note that studies that have empirically determined the differences in hardiness between reproductive and vegetative tissue types often examine one or a few species at a time (Neuner *et al*., 2013; CaraDonna & Bain, 2016; von Büren & Hiltbrunner, 2022; Jin *et al*., 2025). When performing broad, cross‐species analyses aimed at observing patterns in development with hardiness, there is a need to standardize phenology between diverse species to a common scale. Though the BBCH scale is the best existing and most broadly applied scale for making phenological comparisons across species, challenges exist when applying the scale universally to species with different phenological strategies (Finn *et al*., 2007), and their accuracy in describing a given phenological stage is constrained by the subjectivity of the observer (Różańska *et al*., 2025). These challenges become particularly evident in analyses of perennial phenology that include native forest species (such as ours), where precise definitions of phenological stage can be greatly influenced by growth habit (native forest species tend to have larger and more difficult to observe canopies than cultivated fruit trees) or study scale, with reported phenological observations occurring at both individual (Malmqvist *et al*., 2017) and population (Elmendorf *et al*., 2016) levels. Thus, while the standardization of several diverse plant groups to a common phenological scale is necessary, we recognize that it may introduce some limitations in capturing species‐specific phenological nuances.

## Methods of accounting for spring freeze

Thresholds used to quantify spring freeze damage typically fall into two main categories: those that are empirically measured and those that are estimated or model‐derived. Empirically measured thresholds can be further classified by the method in which they are determined, either by field observations of damage following natural spring frosts or through experimentation in controlled conditions:

1. Empirical thresholds: determined from previous field observations or experiments, these thresholds tend to offer greater accuracy for models focused on characterizing damage (Cittadini *et al*., 2006). Empirical determinations of freeze damage usually occur in one of two ways:
  - Experimentation: this approach involves collecting material and exposing it to increasingly negative temperatures in a controlled setting (Proebsting *et al*., 1978). This allows for accurate determination of lethal temperature thresholds, such as LT_50_ (the temperature at which 50% of the tissues are killed). Through this method, thresholds may be determined for material at different phenological stages by collecting material at different times in the field, or forcing (i.e. exposing to warm temperatures) material for different lengths of time in growth chambers (Miranda *et al*., 2005).
  - Observation: this method involves the direct visual inspection of plant tissues after an expected freeze event in the field. The extent of damage is recorded based on visible indicators such as wilted leaves, dead buds, floral abortion, dieback, or tissue oxidation (Harrington, 1936; Wood & Reilly, 2001). While this method provides the advantage of being a direct reflection of field conditions, it does not allow for precise determination of the temperature at which the freezing damage occurs, as temperatures may continue decreasing after the onset of damage, leading to overestimated thresholds. Additionally, this method is dependent on the phenological stage present in the field at the time of frost occurrence.
2. Modeled thresholds: these thresholds rely on generalized assumptions regarding both the plant system and the meteorological conditions of the study site. These thresholds are used when empirical thresholds are not available for the plant of study, or when the scope of the study is too broad for specific thresholds (e.g. a study that has a global scope and considers several species). Therefore, for simplicity, these modeled thresholds tend to be based on broad biological estimates of critical temperatures that injure plant tissues around the equilibrium freezing point of water (Savage *et al*., 2025).

Empirical methods of determining hardiness thresholds in plant tissues provide a direct approach to characterizing freeze risk. Thresholds derived from these studies are ultimately applied to descriptions of hardiness under field conditions and are used to make informed management decisions by relevant parties, such as conservationists and agricultural practitioners (Proebsting *et al*., 1978; Warrington & Rook, 1980). However, reported empirical threshold values should always be considered within the context of how they were determined. Thresholds that are measured in controlled conditions (controlled‐freeze tests) may accurately describe the temperature at which a given plant tissue freezes, but fall short in portraying the highly complex suite of conditions that lead to damage in the field (Fig. 1; Warrington & Rook, 1980). Thresholds reported through field observations of damage are often done so using air temperature as a proxy, which may differ from the actual internal temperature at which tissues freeze (Litschmann & Středa, 2019). Moreover, although empirical determinations of freezing thresholds exist across plant groups, most of the existing work has been performed on cultivated species, where freezing injury has significant economic consequences (Angel, 2007; Lawrimore, 2008; An‐Vo *et al*., 2018).

The use of predictive models to estimate levels of spring freeze damage in the field has become more widespread in recent decades, describing freeze risks in a variety of plant groups (Cittadini *et al*., 2006; Bennie *et al*., 2010; Verocai *et al*., 2022; Qiu *et al*., 2024; Campos‐Arguedas *et al*., 2025; Kim *et al*., 2025). Assessments of freeze risk that arise from these models are directly related to the thresholds that are chosen to estimate damage, and as such, the rationale for choosing a given threshold is central to the efficacy of the model. In some cases, thresholds may be chosen based on previous empirical determinations of hardiness in the given crop of interest (Cittadini *et al*., 2006; Assa *et al*., 2018; Bigler & Bugmann, 2018; Campos‐Arguedas *et al*., 2025). What is more common, however, is the choice of a single threshold that is broadly applied and has either been previously validated, is validated within the scope of the study, or is based on a theoretical understanding of freezing in relation to the microclimate or the equilibrium freezing point of water (Xiao *et al*., 2018; Vitasse *et al*., 2019; Zeng *et al*., 2022; Stuke *et al*., 2024; Kim *et al*., 2025). The chosen threshold may not be directly related to the intrinsic hardiness of the species of interest and can carry substantial uncertainty and lower precision (Savage, 2019), albeit still useful in broadly describing risk. In particular, thresholds that describe critical air temperatures that are greater than empirical determinations of tissue hardiness may be intentionally chosen to avoid underestimating freeze risk. This would account for the effect of microclimatic factors such as radiative cooling, which decreases plant tissue temperature in comparison to air, especially depending on plant height (Fig. 1a; Hoffmann & Rath, 2013; Vitasse *et al*., 2019).

The choice of a freeze damage threshold, whether empirically determined or model‐derived, is often influenced not only by plant species but also by the spatial scale of the study. Broad‐scale studies that include multiple species across large geographic areas commonly apply a generalized threshold for simplicity and comparability (e.g. 0°C in Zeng *et al*. (2022), −2.2°C in Qiu *et al*. (2024)). By contrast, more localized and specific studies may use species‐specific thresholds based on empirical data (Cittadini *et al*., 2006; Qiu *et al*., 2024). Regardless of the method by which they are chosen, disparities in the thresholds used to define spring freeze events can make comparisons across species and studies challenging.

## Quantifying hardiness: exploring trends in reported thresholds

To evaluate broad patterns and discrepancies in freeze temperature thresholds and capture a representative range of plant responses, we analyzed published thresholds (typically the lethal temperature for 50% of tissues (LT_50_)) from empirical (both observational and experimental; *n* = 294) and modeling studies (*n* = 72), grouping species into three categories: native forest (noncultivated trees and shrubs), fruit crops (perennial fruit and nut crops), and row crops. Each data point was further classified by genotype, tissue type (reproductive or vegetative), and the Köppen climate zone(s) of the study location. When phenological stages reported in the original papers were on a different scale, they were recorded as such and standardized to the BBCH scale (Bleiholder *et al*., 2001), allowing for a normalization of developmental time across species and comparisons across studies. Additionally, each study was labeled by its scale (local, regional, or global) and paper type (experimental, modeling, or observational), for cross‐study comparisons of frost sensitivity (Supporting Information Table S1).

## Modeling frost susceptibility across tissue types and phenological stages

Given documented differences in freeze threshold values between vegetative and reproductive tissues (Sakai, 1983; Augspurger, 2013; Ladinig *et al*., 2013; Neuner *et al*., 2013; CaraDonna & Bain, 2016; von Büren & Hiltbrunner, 2022; Jin *et al*., 2025), we have separated our analysis by a tissue type classification, categorizing vegetative and reproductive tissue (largely reflecting leaf‐first and flower‐first strategies for woody perennials, respectively, though not noted in our analyses). For the purpose of this analysis, there is also practical value in separating threshold values based on tissue type. This is primarily due to the nature of the phenological scale utilized (BBCH), which places budbreak and vegetative growth stages (stages 0–39) in distinct categories that precede floral and fruit development (stages 50–99) (Bleiholder *et al*., 2001).

To evaluate the effect of threshold type (empirical vs model) in the reported temperature thresholds (*T*_th_) for either vegetative or reproductive tissue types *i*, we used an exponential decay model. Since observations belonged to two threshold classes, we first fitted a full interaction model using a dummy variable for threshold type (${\mathrm{th}}_{\mathrm{emp}}$) to test potential differences in estimated coefficients between Empirical (${\mathrm{th}}_{\mathrm{emp}}=1$) and Model‐derived (${\mathrm{th}}_{\mathrm{emp}}=0$) thresholds using nonlinear least squares (nls in R):

(Eqn 1) $$T_{{\mathrm{th}}_{i}}=\left\{\begin{array}{c} \left(a+a_{1}\times {\mathrm{th}}_{\mathrm{emp}}\right)\times e^{\left[\left(b+b_{1}\times {\mathrm{th}}_{\mathrm{emp}}\right)\times \left(\text{Stage}\right)\right]}+\left(c+c_{1}\times {\mathrm{th}}_{\mathrm{emp}}\right),\text{for}\;i=\text{vegetative}\\ \left(a+a_{1}\times {\mathrm{th}}_{\mathrm{emp}}\right)\times e^{\left[\left(b+b_{1}\times {\mathrm{th}}_{\mathrm{emp}}\right)\times \left(\text{Stage}-50\right)\right]}+\left(c+c_{1}\times {\mathrm{th}}_{\mathrm{emp}}\right),\text{for}\;i=\text{reproductive} \end{array}\right.$$

where *e* is Euler's number; Stage is the stage of phenological development in the BBCH scale; *a* + *a*_1_ + *c* + *c*_1_ represent the initial intercept at Stage = 0 for *i* = vegetative, or Stage = 50 for *i* = reproductive; *c* + *c*_1_ represent the asymptote of the exponential decay function as Stage increases; and *b* + *b*_1_ represent the decay rate (i.e. the magnitude represents how quickly threshold changes from initial intercept to the asymptote). For both tissue types, we found coefficients *c*_1_, *a*, and *b* to be significant, resulting in the final model:

(Eqn 2) $$T_{{\mathrm{th}}_{i},j}=\left\{\begin{array}{c} a\times e^{\left[b\times \text{Stage}\right]}+\left(c_{1}\times {\mathrm{th}}_{\mathrm{emp}}\right),\;\text{for}\;i=\text{vegetative}\\ a\times e^{\left[b\times \left(\text{Stage}-50\right)\right]}+\left(c_{1}\times {\mathrm{th}}_{\mathrm{emp}}\right),\;\text{for}\;i=\text{reproductive} \end{array}\right.$$

## Spring freeze risk in vegetative and reproductive tissues: trends and considerations

**Fig. 2:**
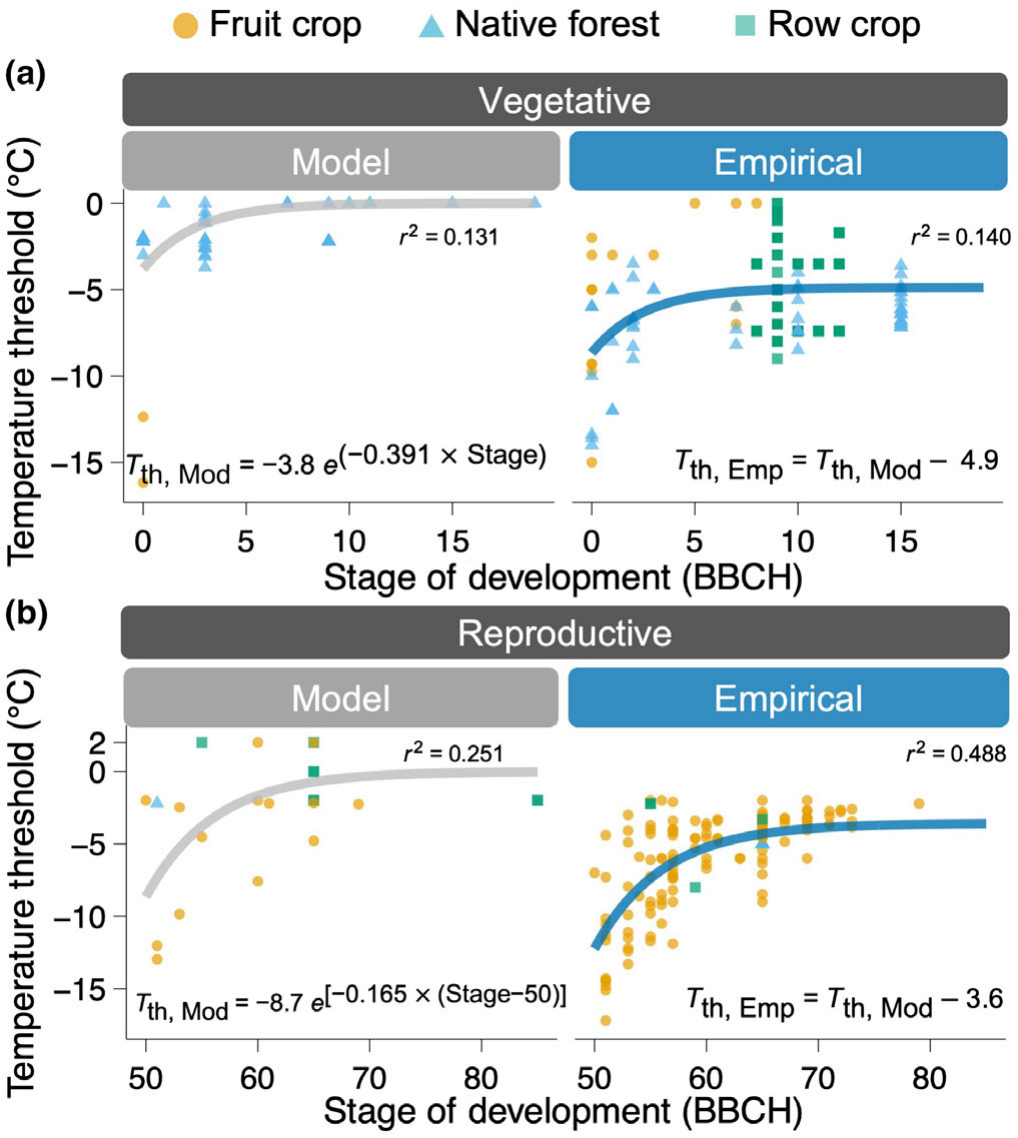
Modeled and empirical temperature thresholds for spring freeze damage across phenological development stages compiled from 53 studies. Thresholds for freeze damage are shown for (a) vegetative tissues (BBCH 0–15; *n =* 195) and (b) reproductive tissues (BBCH 50–85, *n =* 171). Species were separated into three groups: fruit crop, native forest, and row crop (for the full list, see Supporting Information Table S1). Lines are nonlinear least squares fits for each threshold type in response to the stage of development. Models and associated pseudo‐*R* ^2^ are shown within each panel.

For both reproductive and vegetative tissues, hardiness to spring freeze events is lost nonlinearly as development progresses (Fig. 2). The resulting models show that although modeled and empirical temperature thresholds decrease at the same rate over development, the significant coefficient *c*_1_ results in a constant difference between thresholds throughout development (−4.9 ± 0.3°C for vegetative and −3.6 ± 0.4°C for reproductive). This difference was expected, as air temperature tends to overestimate plant tissue temperatures – especially during radiative freezes. However, the difference found here for vegetative tissues is larger than some of the largest reported differences for station to ground level (e.g. 4.1°C; Sentelhas *et al*., 1995). For reproductive tissue, the difference of −3.6°C is within that range. However, fruit trees – from which most of our data are derived – are generally of similar height as weather stations (Fig. 1), and have smaller differences between measured tissue and air temperature (Peña Quiñones *et al*., 2019).

Though some evidence has suggested that newly growing tissues regain some level of hardiness as they mature (Taschler *et al*., 2004; Neuner, 2014; Vitasse *et al*., 2014), the high levels of cold hardiness observed before budbreak or in early stages post‐budbreak (*c*. 00–03, or 50–55) are not regained. Due to differences in the timing of phenology across species (Hodgson *et al*., 2011; Wolkovich *et al*., 2014; Kovaleski, 2022), it is unlikely that such regain would appear in an analysis across species. Thus, at the modeling level, we can only observe that both vegetative and reproductive tissues lose cold hardiness upon emergence and remain at that level. However, when evaluating risk, the tissues are most vulnerable when newly emerged, as the likelihood of occurrence of spring freezes is higher earlier in the season (Vitasse *et al*., 2014; CaraDonna & Bain, 2016). The loss of hardiness associated with phenological development is coupled with an apparent convergence across species to a common minimum value, though this value itself differs between vegetative and reproductive tissues (Fig. 2).

It is important to note that estimating tissue‐level susceptibility as LT_50_ (50% damage) may overestimate whole‐tree impacts under moderate freeze conditions. This is partly due to variability within an individual, as buds on the same tree often span multiple phenophases (Milla *et al*., 2010; Vander Mijnsbrugge & Moreels, 2020). Larger perennials can also recover by activating blind nodes or unbroken buds (Friend *et al*., 2011). Such heterogeneity can be further modulated by carbon and water status within individual branches, as demonstrated by girdling and defoliation experiments that altered branch‐specific cold hardiness (Poirier *et al*., 2010). Incorporating this variability and the recovery potential of perennial plants is therefore a key future step toward more biologically realistic freeze‐risk projections.

While numerous studies have established that reproductive tissues are generally more vulnerable to freeze events compared to vegetative tissues (Ladinig *et al*., 2013; Neuner, 2014; CaraDonna & Bain, 2016; von Büren & Hiltbrunner, 2022; Jin *et al*., 2025), other research suggests that certain species or specific developmental stages may exhibit comparable or even enhanced hardiness in reproductive structures (Augspurger, 2013; Bertel *et al*., 2021). Our analysis aligns more closely with the latter findings, indicating that reproductive tissues with springtime emergence may exhibit comparable or even slightly greater hardiness than vegetative tissues at the earliest phenological stages, though this difference diminishes as development progresses (Fig. 2a,b). This may be due to temporary physiological advantages that favor early reproductive development (Neuner *et al*., 2013) reflecting species‐specific spring freeze hardiness strategies related to ecological advantages (CaraDonna & Bain, 2016).

The physiological basis underpinning differences in reported hardiness thresholds between tissues may be explained by extending the Flower Economic Spectrum (FES) to spring freeze tolerance. The FES describes how flowers vary in traits such as size, longevity, and construction/maintenance costs (Roddy *et al*., 2021). Under this expanded perspective, floral buds, typically more fragile and costly (e.g. larger surface area, higher metabolic maintenance) (Neuner *et al*., 2013; Roddy *et al*., 2021), exhibit greater freeze hardiness early in development as part of an evolutionary trade‐off prioritizing reproductive success, particularly during narrow pollination windows, but show a sharp decrease in freeze hardiness as they develop (Savage & Cavender‐Bares, 2013; CaraDonna & Bain, 2016; Gaudinier & Blackman, 2020; Savage *et al*., 2025). By contrast, vegetative buds are generally less expensive, more robust, and structured for long‐term survival, suggesting that it may be advantageous for vegetative tissues to retain greater hardiness to freeze events in both early and late stages of development (e.g. smaller difference between the intercept and asymptote values) (Neuner *et al*., 2013; Wagner *et al*., 2021). Our analysis suggests that, based on the FES framework, early‐stage reproductive buds may be more structurally or metabolically prepared to withstand frost events – perhaps as a strategy to secure reproductive potential under uncertain earlier spring conditions – before shifting toward a more resource‐intensive, vulnerable phase later in their development when lower environmental risk of low temperatures is present.

Though the analysis presented here represents a broad range of plant groups, each group is not equally represented by tissue type. For example, patterns of hardiness thresholds in reproductive tissues are primarily represented through studies in fruit trees, while studies focused on vegetative tissue favor native forest species (Fig. 2). The ‘skew’ of different plant groups to different tissue types is likely a consequence of the perceived relevance of each tissue type to respective plant groups: reproductive tissues in cultivated fruit trees are agronomically essential and directly economically important compared to their vegetative counterparts, whereas vegetative tissues in native species are emphasized over reproductive tissues because of their ecological contributions to primary production (Savage *et al*., 2025). Thus, comparing tissue‐type hardiness across plant groups remains challenging given uneven representation in the existing literature. To assess potential influences beyond tissue type, we analyzed subsets of the dataset by ecological and biogeographical groupings, such as deciduous vs evergreen, early vs late phenology, and North American vs European species, among others (Figs S1, S2). When present, only minor differences exist between groups, and the most consistent pattern remains the contrast between modeled and empirical thresholds (Table S2). This distinction is particularly relevant given the limited empirical data on spring freeze damage to reproductive structures in native forest species – represented here by just two studies (Qiu *et al*., 2024; Savage *et al*., 2025). Cold damage to reproductive tissues during early development results in reduced carbon allocation toward reproduction within a season. We speculate that increased carbon storage from failed reproductive years can contribute to observed masting behavior in years with favorable spring conditions (Piovesan & Adams, 2005; Vacchiano *et al*., 2021). We also identify that the lack of species comparisons related to flower‐first vs leaf‐first could also return differences in thresholds within reproductive or vegetative parts – though we did not include those as part of our analysis.

## Model derived and empirically determined thresholds: disjuncts and consequences

Comparisons of thresholds within tissue types reveal a significant difference in baseline hardiness thresholds used between those that are empirically determined and model‐derived. While the rate of hardiness loss in relation to developmental stages does not differ between threshold types, empirically derived thresholds are consistently lower than those that are derived from models (Fig. 2). This intercept difference (meaning a constant difference across the development), regardless of tissue type, suggests that modeled thresholds systematically overestimate spring freeze damage (Marquis *et al*., 2022). In our analysis, the empirical least cold hardiness for vegetative tissues as they develop is −4.9 ± 0.3°C, whereas that value is 0°C (or −0.2 ± 0.7°C if coefficient *c* is retained in Eqn 1) for modeled thresholds (Fig. 2a). Similarly, for reproductive tissues, the empirical least cold hardiness is −3.6 ± 0.4°C, while that value is 0°C (or −0.5 ± 0.4°C if coefficient *c* is retained in Eqn 1) for modeled thresholds (Fig. 2b).

Though differences in thresholds exist between those that are empirically determined and those that are model‐derived, they are generally smaller in studies considering reproductive tissue (Fig. 2a). This is likely because there is a greater body of research concerned with empirical determinations of reproductive freeze damage, skewed heavily in favor of economically significant fruit and nut crops (Savage *et al*., 2025) where the survival of reproductive structures is directly related to economic outputs. These aspects are also reflected in our collection of studies used to generate the dataset analyzed here (Table S1). Because there is a greater awareness of empirical determinations of hardiness in reproductive tissues of these plants, they are more often incorporated into models as thresholds used to estimate spring freeze damage (e.g. models in Cittadini *et al*. (2006); Assa *et al*. (2018)).

## Considering the context of a warming climate

Here, temperature thresholds used to describe hardiness to spring freeze events in plants are primarily examined as a function of phenological stage, tissue type, and the method by which the threshold is derived (empirical measurement or modeling). However, other points of consideration are likely also relevant. On an intraspecific level, temperatures at which tissues freeze are likely to differ across climatologically variable locations. Although noted for each study (Table S1), effects of Köppen climate classification in determining temperature thresholds were not explicitly analyzed in this review, as a more diverse representation of climates would be necessary to elucidate any significant relationships. However, factors that influence climate definition, such as humidity, wind speed, topography, altitude, and cloud cover, are all relevant factors when exploring and determining thresholds for freeze damage in a given location, since they affect the occurrence of freezes (Fig. 1; Bill *et al*., 1979; Blennow & Persson, 1998; Logan *et al*., 2000; Charrier *et al*., 2015; Wang *et al*., 2025).

Beyond abiotic variables, plant structure and physiology may also play a role in how environmental conditions can cause tissue damage. For example, bud position within the canopy, pubescence, or the presence of bud scales can buffer tissues against temperature drops (Smith, 1974). Tissue hydration, osmotic potential, and concentrations of compounds such as sugars, carbohydrates, and proteins could also interact with the environment and potentially contribute to spring freeze hardiness strategies (Gusta *et al*., 2004; Charrier *et al*., 2015; Hajihashemi *et al*., 2020; Ghosh *et al*., 2021). Therefore, as climate change alters the conditions to which plants are exposed (Román‐Palacios & Wiens, 2020; Marquis *et al*., 2022), we can expect that thresholds may need to be adapted as climates shift.

In a climate change context, freeze risk is a dynamic process as warming advances both phenology (Kramer *et al*., 2000; Inouye, 2008; Vitasse *et al*., 2011; Clark *et al*., 2014; Piao *et al*., 2019; Chen *et al*., 2024; Hassan *et al*., 2024), and date of last freeze (Easterling, 2002; Zhong *et al*., 2017; Masaki, 2021; Wang *et al*., 2024). Therefore, it is the interaction of both that will define the changes in the length of the window for spring freeze damage and its shift in time. Considering that greater climatic variability is an inherent consequence of long‐term climate change (Bhatti *et al*., 2024), increased frequency of late‐season cold spells may increase occurrences of damage, even if years are warmer on average (Francis & Skific, 2015; Casson *et al*., 2019; Cohen *et al*., 2023). When simulating the ecological and agronomical implications of future warming scenarios on plant survival and long‐term viability, these factors should be considered to ‘bridge the gap’ between the physiological mechanisms underlying hardiness to spring freezes and their interaction with broader climatological factors. At a basic level, this could mean incorporating phenology models in evaluations of spring freeze damage given the strong effect of development on damage thresholds (Fig. 2).

## Conclusions

In temperate and boreal ecosystems, late spring frosts are not unusual, but their impact is becoming increasingly critical. While overall temperatures are projected to increase with climate change, the risk of spring freeze is simultaneously increasing due to advancements in spring phenology and greater severity and frequency of low‐temperature variability. Thus, determining the risks of late spring frost damage is of particular interest for a wide array of plants and plant tissues.

More comprehensive empirical research is needed to validate and improve existing models, particularly regarding responses to spring freeze events of vegetative tissues more broadly, and reproductive tissues of forest species. Current discrepancies between model‐derived and empirically determined spring freeze thresholds highlight the need for better integration of physiological and phenological data. An integration of physiological data reflective of a deeper understanding of plant–environmental interactions within models will only become more relevant in providing accurate predictions of plant responses to future climatic change. Additionally, though there have been attempts to provide standardized classifications of phenological stages, there are still challenges associated with the development and application of a universal phenological scale, due in large part to the different phenological strategies that have evolved among plant groups. For broad‐scale models aimed at describing freeze risk and its consequences, improvement in the standardization of phenological scales, or in enhancing translatability between scales, could allow for stronger estimations of freeze risk in relation to development.

As climate change continues to affect spring onset and spring freeze patterns, understanding and acknowledging their occurrence becomes crucial for predicting and managing spring freeze risk in natural and managed ecosystems. Having a holistic approach and integrating this knowledge will be key for understanding species adaptation potential for developing effective conservation strategies in response to the accelerated environmental change. To meet these challenges, based on the literature reviewed here and on our analyses of published thresholds, we provide recommendations for research involving spring freezes in Box 1.

Box 1 **Recommended practices and future directions for studies evaluating spring freeze events**

1. When possible, studies evaluating spring freeze hardiness in relation to phenology should use phenological scales that have been applied broadly in existing studies, such as BBCH. Continued work to apply these scales to a broader range of plant groups, particularly noncultivated plants, is also needed for consistent cross‐species comparisons.
2. Studies using air temperature as a proxy for describing hardiness to spring freeze should evaluate microclimatic factors that could contribute to discrepancies between measured air temperature and the tissue temperature at which spring freeze damage occurs, such as humidity, wind speed, cloud cover, topography, and plant height, and incorporate these factors into models when applicable.
3. When modeling spring freeze risk, consider and acknowledge possible disjuncts such as overestimations of risk that may occur from applying a broad threshold to the study system of interest.
4. Studies aimed at empirical determinations of spring freeze hardiness thresholds should expand to include (1) more noncultivated and ecologically diverse plant groups, such as native forest species, and (2) tissue types not traditionally studied in a given plant group (particularly vegetative tissues in cultivated plants, and reproductive tissues in native species).
5. Future work is needed to evaluate how differing phenological strategies influencing developmental timing in perennial plants, such as flower‐first vs leaf‐first development, contribute to spring freeze hardiness and risk depending on timing.
6. Long‐term studies monitoring the impacts of spring freeze events on both natural ecosystems and agroecosystems in a diverse range of climates should both continue and expand in order to better inform models predicting these effects for future climate warming scenarios.
7. Incorporation of phenology and the developmental stages of young, emerging tissues is needed to provide accurate predictions of freeze damage and its long‐term ecological and economic consequences from local to global scales.

Limitations exist in all studies, and particularly incorporating recommendations 1–3 may not be possible. However, we suggest that stating assumptions made in different aspects of analyses related to these recommendations can help with standardization and comparability of studies.

## Competing interests

None declared.

## Author contributions

EK, FC‐A, NSA and APK conceived this review. EK and FC‐A collected, prepared, and analyzed data under the supervision of APK. EK and FC‐A drafted the initial manuscript, with reviews and contributions from NSA and APK. EK and FC‐A contributed equally to this work.

## Disclaimer

The New Phytologist Foundation remains neutral with regard to jurisdictional claims in maps and in any institutional affiliations.

## Supporting information


**Fig. S1** Modeled and empirical temperature thresholds for spring freeze damage across phenological development stages.
**Fig. S2** Empirical temperature thresholds for spring freeze damage across phenological development stages.
**Notes S1** Threshold variation across ecological and biogeographical groups.

**Table S1** Reported thresholds used to characterize spring freeze risk.
**Table S2** Parameter estimates from subsets of the full dataset, grouped by different factors.
Please note: Wiley is not responsible for the content or functionality of any Supporting Information supplied by the authors. Any queries (other than missing material) should be directed to the *New Phytologist* Central Office.

## Data Availability

All data used in this manuscript are available in Table S1.
