# Supplementary material for: Thresholds for spring freeze: measuring risk to improve predictions in a warming world
Source: New Phytol. 2025 Aug 7;248(2):563–75. doi: 10.1111/nph.70453 (PMC12445811; doi:10.1111/nph.70453)
Supplement: Supplementary file 1 — Fig. S1 Modeled and empirical temperature thresholds for spring freeze damage across phenological development stages. Fig. S2 Empirical temperature thresholds for spring freeze damage across phenological development stages. Notes S1 Threshold variation across ecological and biogeographical groups. [file NPH-248-563-s001.pdf]

New Phytologist

***New Phytologist* Supporting Information**

Article title: Thresholds for spring freeze: measuring risk to improve predictions in a warming world
Authors: Erica Kirchhof, Francisco Campos-Arguedas, Nadia Soledad Arias, Al P. Kovaleski
Article acceptance date: 14 July 2025

The following Supporting Information is available for this article:

Notes S1 Threshold variation across ecological and biogeographical Groups

Fig. S1 Modeled and empirical temperature thresholds for spring freeze damage across phenological development stage.
Fig. S2 Empirical temperature thresholds for spring freeze damage across phenological development stage.

Table S1 Reported thresholds used to characterize spring freeze risk (in a separate file).
Table S2 Parameter estimates from subsets of the full dataset, grouped by different factors.

Notes S1 Threshold Variation Across Ecological and Biogeographical Groups

To assess potential influences beyond tissue type, we analyzed subsets of the full dataset based on ecological and biogeographical groupings, such as early vs. late phenology, deciduous vs. evergreen, bushy berry fruits and tree fruits (**Fig. S1**) and North American vs. European species (**Fig. S2;** see **Table S1** for individual classifications).

Within each grouping, we fitted an interaction model using dummy variables for threshold type (th_{emp}) and group category ($grouping_j$) to test for differences in estimated coefficients between empirical ($th_{emp} = 1$) and model-derived ($th_{emp} = 0$) thresholds, as well as across the defined group categories (using $th_{group} = 1$, or $th_{group} = 0$ for each comparison). The model was fitted using nonlinear least squares (nls in R) using the following equation:

$$\mathrm{T}_{th} = \mathrm{a} \times e^{\mathrm{b} \times Stage} + (\mathrm{c} \cdot \mathrm{th_{emp}} + \mathrm{c_1} \cdot th_{group});$$

where *e* is Euler's number; *Stage* is the stage of phenological development in the BBCH scale; $a+c+c_1$ represent the initial intercept at *Stage*=0; $c+c_1$ represent the additive effect of threshold type (th_{emp}) and grouping (th_{group}) on the asymptotic value of the exponential decay function as *Stage* increases; and *b* represent the decay rate (i.e., the magnitude represents how quickly threshold changes from initial intercept to the asymptote). For bushy berry fruits, and for continent of origin analyses, modeled thresholds were not present or insufficient, and therefore a model was not fit.

Across all groupings, the *c* parameter, which differentiates between modeled-derived and empirical thresholds, was significant. In contrast, parameter c_1, which reflects the differences in thresholds between the ecological and biogeographical groups was not significant for early vs. late phenology, deciduous vs. evergreen, bushy berry fruits and tree fruits, indicating there are no differences between thresholds for these groups (**Table S2**). For continental comparisons, marginal differences were observed between North American and European species, both for all genera combined and for species within the genus *Prunus*. However, the magnitude of these differences was relatively small (0.82 ºC and 1.38ºC, respectively), indicating little biological relevance (**Table S2**).

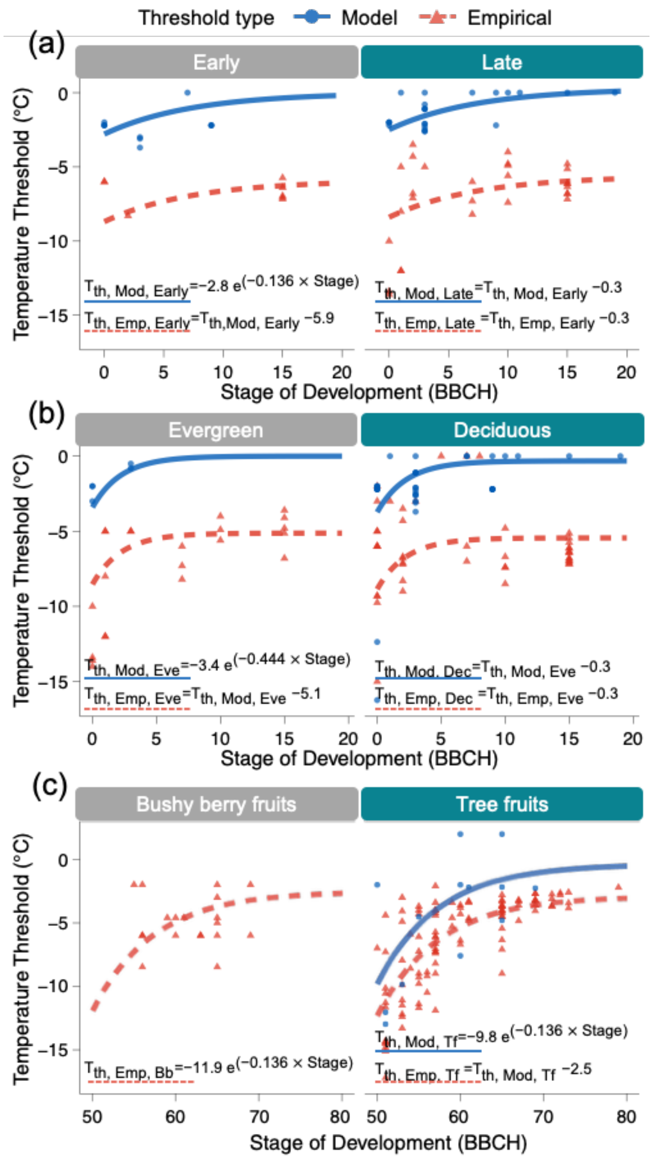

Fig. S1 Modeled (solid line) and empirical (dashed line) temperature thresholds for spring freeze damage across phenological development stage. Thresholds for freeze damage for **(a)** vegetative tissues (BBCH 0–15; *n=70*) grouped by early vs late spring phenology, **(b)** vegetative tissues (BBCH 0–15; *n=109*) grouped by their habit (evergreen vs deciduous), and **(c)** reproductive tissues (BBCH 50–85, *n=137*) grouped by bushy berry fruits and tree fruits. Lines are non-linear square fits for each threshold type within each categorical grouping in response to stage of development.

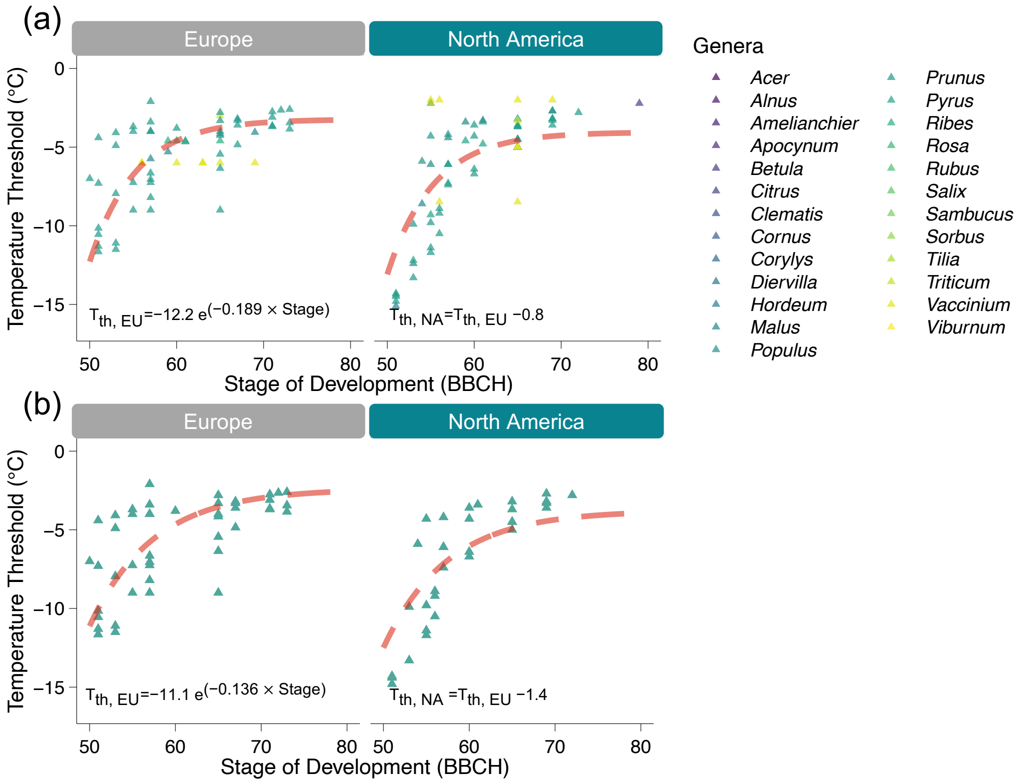

Fig. S2 Empirical temperature thresholds for spring freeze damage across phenological development stage. Thresholds for freeze damage for **(a)** reproductive tissues (BBCH 50–85, *n=141*) from all genera grouped by continent of origin, and **(b)** reproductive tissues (BBCH 50–85, *n*=77) from the genus *Prunus* grouped by continent of origin. Lines represent non-linear square fits of empirical thresholds within each categorical group based on developmental stage.

Table S2 Parameter estimates from subsets of the full dataset, grouped by different factors. The estimated coefficient (c_1) is reported with its standard error for each grouping factor. Sample sizes (*n*) reflect the number of species or data points in each subset.

Grouping factor	c_1 (group term)	*P*-value	Interpretation*
Deciduous vs. evergreen *(vegetative, n= 70)*	–0.32 ± 0.46	0.49	ns
Early- vs. late-phenology (forest species) *(vegetative, n= 109)*	+0.30 ± 0.48	0.54	ns
Bushy berries vs. tree fruits *(reproductive, n= 137)*	–0.39 ± 0.55	0.48	ns
Continent (all genera) *(reproductive, n= 141)*	–0.82 ± 0.36	0.03	significant effect, low biological relevance
Continent (*Prunus* only) *(reproductive, n=77)*	–1.38 ± 0.53	0.01	significant effect, low biological relevance

**Interpretation is based on the P-values obtained through t-tests of each estimated coefficient, with "ns" indicating non-significance (p > 0.05).*
